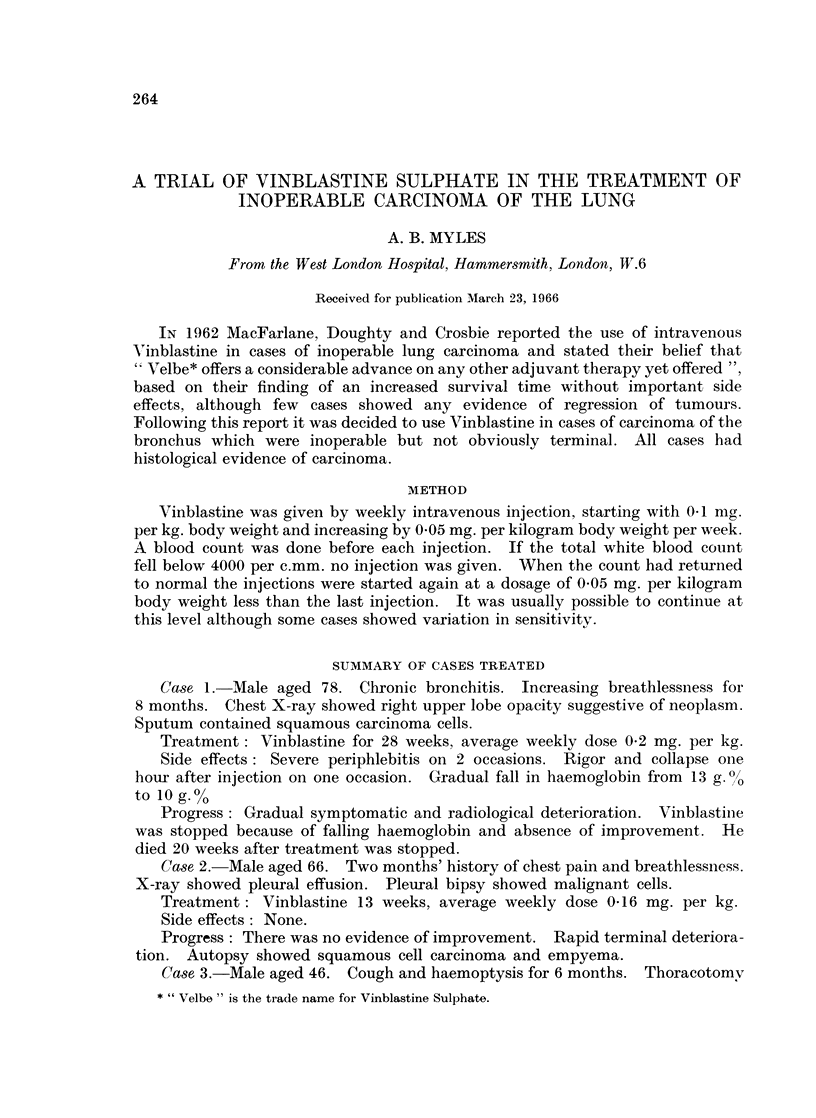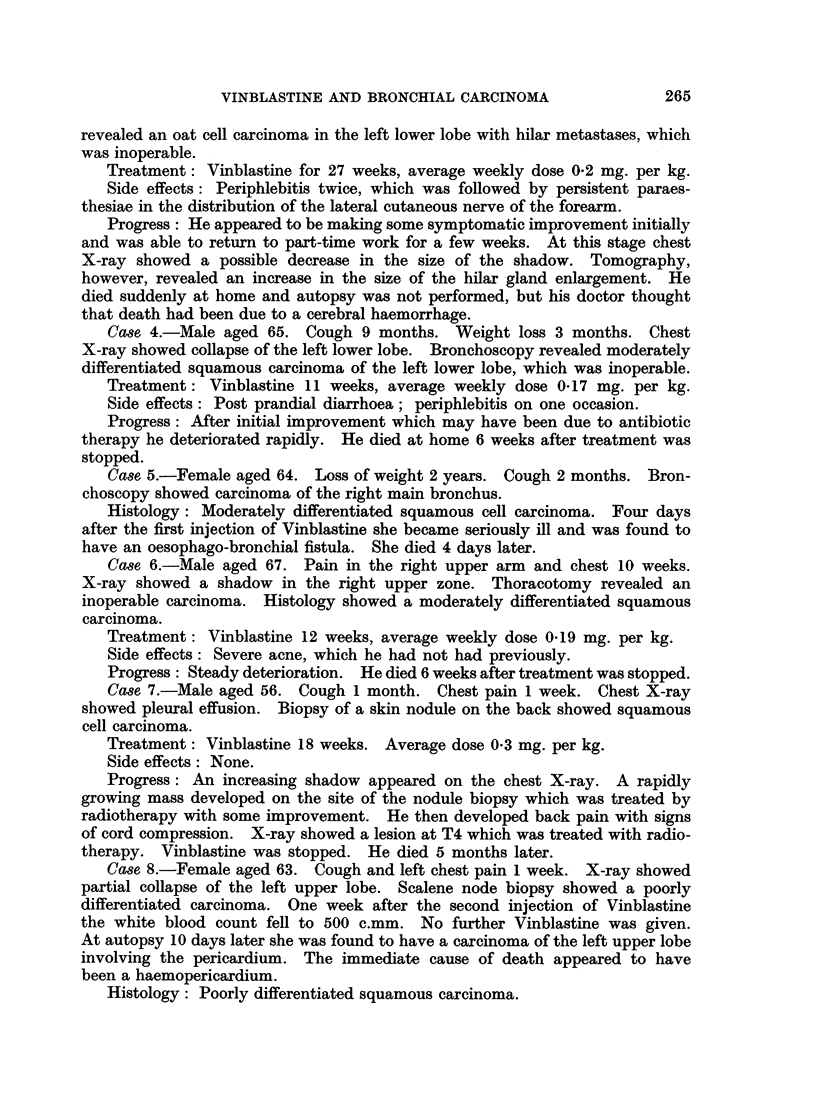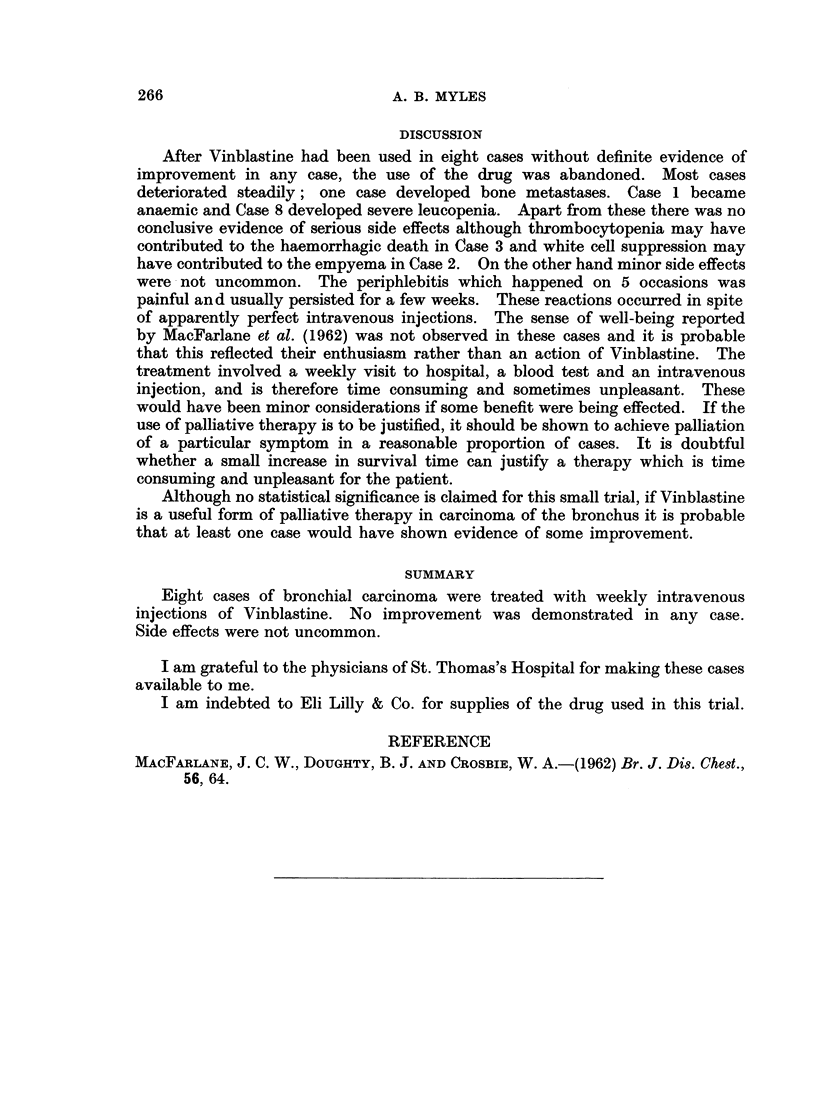# A trial of vinblastine sulphate in the treatment of inoperable carcinoma of the lung.

**DOI:** 10.1038/bjc.1966.33

**Published:** 1966-06

**Authors:** A. B. Myles


### A TRIAL OF VINBLASTINE SULPHATE IN THE TREATMENT OF INOPERABLE CARCINOMA OF THE LUNG

A. B. MYLES

*From the West London Hospital, Hammersmith, London, W.6*

Received for publication March 23, 1966

IN 1962 MacFarlane, Doughty and Crosbie reported the use of intravenous Vinblastine in cases of inoperable lung carcinoma and stated their belief that " Velbe* offers a considerable advance on any other adjuvant therapy yet offered ", based on their finding of an increased survival time without important side effects, although few cases showed any evidence of regression of tumours. Following this report it was decided to use Vinblastine in cases of carcinoma of the bronchus which were inoperable but not obviously terminal. All cases had histological evidence of carcinoma.

### METHOD

Vinblastine was given by weekly intravenous injection, starting with 0·1 mg. per kg. body weight and increasing by 0·05 mg. per kilogram body weight per week. A blood count was done before each injection. If the total white blood count fell below 4000 per c.mm. no injection was given. When the count had returned to normal the injections were started again at a dosage of 0·05 mg. per kilogram body weight less than the last injection. It was usually possible to continue at this level although some cases showed variation in sensitivity.

### SUMMARY OF CASES TREATED

*Case* 1.—Male aged 78. Chronic bronchitis. Increasing breathlessness for 8 months. Chest X-ray showed right upper lobe opacity suggestive of neoplasm. Sputum contained squamous carcinoma cells.

Treatment : Vinblastine for 28 weeks, average weekly dose 0·2 mg. per kg.

Side effects : Severe periphlebitis on 2 occasions. Rigor and collapse one hour after injection on one occasion. Gradual fall in haemoglobin from 13 g.% to 10 g.%

Progress : Gradual symptomatic and radiological deterioration. Vinblastine was stopped because of falling haemoglobin and absence of improvement. He died 20 weeks after treatment was stopped.

*Case* 2.—Male aged 66. Two months' history of chest pain and breathlessness. X-ray showed pleural effusion. Pleural bipsy showed malignant cells.

Treatment : Vinblastine 13 weeks, average weekly dose 0·16 mg. per kg.

Side effects : None.

Progress : There was no evidence of improvement. Rapid terminal deterioration. Autopsy showed squamous cell carcinoma and empyema.

*Case* 3.—Male aged 46. Cough and haemoptysis for 6 months. Thoracotomy

* " Velbe " is the trade name for Vinblastine Sulphate.

revealed an oat cell carcinoma in the left lower lobe with hilar metastases, which was inoperable.

Treatment : Vinblastine for 27 weeks, average weekly dose 0·2 mg. per kg.

Side effects : Periphlebitis twice, which was followed by persistent paraesthesiae in the distribution of the lateral cutaneous nerve of the forearm.

Progress : He appeared to be making some symptomatic improvement initially and was able to return to part-time work for a few weeks. At this stage chest X-ray showed a possible decrease in the size of the shadow. Tomography, however, revealed an increase in the size of the hilar gland enlargement. He died suddenly at home and autopsy was not performed, but his doctor thought that death had been due to a cerebral haemorrhage.

*Case* 4.—Male aged 65. Cough 9 months. Weight loss 3 months. Chest X-ray showed collapse of the left lower lobe. Bronchoscopy revealed moderately differentiated squamous carcinoma of the left lower lobe, which was inoperable.

Treatment : Vinblastine 11 weeks, average weekly dose 0·17 mg. per kg.

Side effects : Post prandial diarrhoea ; periphlebitis on one occasion.

Progress : After initial improvement which may have been due to antibiotic therapy he deteriorated rapidly. He died at home 6 weeks after treatment was stopped.

*Case* 5.—Female aged 64. Loss of weight 2 years. Cough 2 months. Bronchoscopy showed carcinoma of the right main bronchus.

Histology : Moderately differentiated squamous cell carcinoma. Four days after the first injection of Vinblastine she became seriously ill and was found to have an oesophago-bronchial fistula. She died 4 days later.

*Case* 6.—Male aged 67. Pain in the right upper arm and chest 10 weeks. X-ray showed a shadow in the right upper zone. Thoracotomy revealed an inoperable carcinoma. Histology showed a moderately differentiated squamous carcinoma.

Treatment : Vinblastine 12 weeks, average weekly dose 0·19 mg. per kg.

Side effects : Severe acne, which he had not had previously.

Progress : Steady deterioration. He died 6 weeks after treatment was stopped.

*Case* 7.—Male aged 56. Cough 1 month. Chest pain 1 week. Chest X-ray showed pleural effusion. Biopsy of a skin nodule on the back showed squamous cell carcinoma.

Treatment : Vinblastine 18 weeks. Average dose 0·3 mg. per kg.

Side effects : None.

Progress : An increasing shadow appeared on the chest X-ray. A rapidly growing mass developed on the site of the nodule biopsy which was treated by radiotherapy with some improvement. He then developed back pain with signs of cord compression. X-ray showed a lesion at T4 which was treated with radiotherapy. Vinblastine was stopped. He died 5 months later.

*Case* 8.—Female aged 63. Cough and left chest pain 1 week. X-ray showed partial collapse of the left upper lobe. Scalene node biopsy showed a poorly differentiated carcinoma. One week after the second injection of Vinblastine the white blood count fell to 500 c.mm. No further Vinblastine was given. At autopsy 10 days later she was found to have a carcinoma of the left upper lobe involving the pericardium. The immediate cause of death appeared to have been a haemopericardium.

Histology : Poorly differentiated squamous carcinoma.

DISCUSSION

After Vinblastine had been used in eight cases without definite evidence of improvement in any case, the use of the drug was abandoned. Most cases deteriorated steadily; one case developed bone metastases. Case 1 became anaemic and Case 8 developed severe leucopenia. Apart from these there was no conclusive evidence of serious side effects although thrombocytopenia may have contributed to the haemorrhagic death in Case 3 and white cell suppression may have contributed to the empyema in Case 2. On the other hand minor side effects were not uncommon. The periphlebitis which happened on 5 occasions was painful and usually persisted for a few weeks. These reactions occurred in spite of apparently perfect intravenous injections. The sense of well-being reported by MacFarlane *et al.* (1962) was not observed in these cases and it is probable that this reflected their enthusiasm rather than an action of Vinblastine. The treatment involved a weekly visit to hospital, a blood test and an intravenous injection, and is therefore time consuming and sometimes unpleasant. These would have been minor considerations if some benefit were being effected. If the use of palliative therapy is to be justified, it should be shown to achieve palliation of a particular symptom in a reasonable proportion of cases. It is doubtful whether a small increase in survival time can justify a therapy which is time consuming and unpleasant for the patient.

Although no statistical significance is claimed for this small trial, if Vinblastine is a useful form of palliative therapy in carcinoma of the bronchus it is probable that at least one case would have shown evidence of some improvement.

SUMMARY

Eight cases of bronchial carcinoma were treated with weekly intravenous injections of Vinblastine. No improvement was demonstrated in any case. Side effects were not uncommon.

I am grateful to the physicians of St. Thomas's Hospital for making these cases available to me.

I am indebted to Eli Lilly & Co. for supplies of the drug used in this trial.

REFERENCE

MacFarlane, J. C. W., Doughty, B. J. and Crosbie, W. A.—(1962) *Br. J. Dis. Chest.*, **56**, 64.